# Ulrich Engelmann, Martin Baumann: Zielführend moderieren. Kompetenzen – Methoden – Wege zum Gesprächserfolg

**DOI:** 10.3205/zma001549

**Published:** 2022-07-15

**Authors:** Sigrid Harendza

**Affiliations:** 1 Universitätsklinikum Hamburg-Eppendorf, III. Medizinische Klinik, Hamburg, Germany

## Bibliographical details

Ulrich Engelmann, Martin Baumann

**Zielführend moderieren. Kompetenzen – Methoden – Wege zum Gesprächserfolg**

UTB GmbH

Year of publication: 2022, 438 pages, prize: € 34,90

ISBN: 978-3825256890

## Review

Do you need to moderate? Then this book will most likely become your friend and companion. Are you already a moderation professional? Again, the authors have ensured that you will discover new things and view or brush up on familiar ones from a new perspective. This book is so much more than a guidebook. Already in the preface and in the “Notes on this book” it becomes clear what is important to the authors: the individual possibility of using what has been read and the self-reflection process of the reader. One can either, after a short self-test for one's own classification (novice, adept, professional), be taken on a journey through the book by the corresponding characters, all of whom are humorously illustrated. This is motivating and whets the appetite for the subject. Or one can orient oneself quite classically with an excellently structured and, if necessary, also – depending on one's self-assessment – individualized table of contents. This allows, for example, to find out specifically about certain moderation methods, e.g. for getting to know each other in the group or obtaining feedback. Orientation is additionally facilitated by the drawn companions and by color coding, depending on what is most appealing to the reader.

In general, one is always addressed while reading and feels individually cared for and valued – exactly how one should behave with the individuals of the entrusted group when moderating. In addition to the actual execution of a moderation as well as various moderation methods, which are very well explained with tips and tricks to try out, the book embeds the purely technical aspects of moderation in the contexts of group dynamics, change of perspective and leadership. After all, one is dealing with people and therefore inevitably comes into contact with aspects from these areas. Due to clever cross-referencing in the text, one can leave the originally chosen path and further explore one of these meta-level topics if one wishes. Important group dynamic aspects such as “disturbances have priority” always come into focus in this way, as they can torpedo the best moderation method.

In addition, the basic structure of moderation as well as various techniques are outlined in clear illustrations and diagrams in different styles; their use and effects are referred to in the last chapter. Thus, in addition to moderation and many aspects of cognitive psychology, for which the scientific references are given in case of further interest, one can also learn something about graphical presentation possibilities at the same time. In general, the evidence is well selected and placed. Moreover, useful references in this book are never lecturing, but presented in a pleasant way, appreciative and really helpful, even if they are actually self-evident things, which, however, even elapse professional moderators sometimes. For example, that the moderating person's project is the group. Or, that one should focus on the solutions and not on the problems. And, that it is worth checking before a workshop to see if the flip chart stand is wobbly or the markers are empty. Particular blunders that moderators can fall into and how to avoid them are marked with a caution sign at the margin, making them easier to find if one needs to quickly look something up again. Basic assumptions about human communication that sometimes get lost in heated debates are also brought to mind - in some places as if by accident in footnotes (“Always assume that other people are okay to begin with.”). 

But there is nothing accidental in this book. The topics and contexts are extremely well thought out and researched. Everything is meant to be as enjoyable as possible when reading. This also includes the fact that the book is available both as a hardcover version and as a digital version, which also takes into account the readers’ preferences. In keeping with the increasing digitalization, there is also a chapter on online moderation – in both versions, of course. Most importantly, however, the authors see themselves as learners and very artfully moderate the shift from factual to meta-level and back again in their book – therein lies the skill to be demonstrated in moderation. They articulate expectations for readers while encouraging them at the same time to provide feedback on whether the book contributed to their individual implementation of moderation. My conclusion: the most enjoyable and educational specialist book I have read in a long time.

## Competing interests

The author declares that she has no competing interests.

